# Ultrasound-guided needle release of A1 pulley combined with corticosteroid injection is more effective than ultrasound-guided needle release alone in the treatment of trigger finger

**DOI:** 10.1186/s12893-022-01665-1

**Published:** 2022-06-07

**Authors:** Yan-Yan Wu, Kai Chen, Fan-Ding He, Jie-Rong Quan, Xuan-Yan Guo

**Affiliations:** Department of Ultrasonic, Sichuan Academy of Medical Sciences & Sichuan Provincal People’s Hospital, School of Medicine, University of Electronic Scienceand Technology of China, No. 32, West Second Section, First Ring Road, Qingyang District, Chengdu, 610072 Sichuan China

**Keywords:** A1 pulley, Corticosteroid injection, Trigger finger, Ultrasonography-guided release

## Abstract

**Background:**

The purpose of the present study was to evaluate the clinical effectiveness of ultrasonography-guided needle release of A1 pulley combined with corticosteroid injection by comparing it with ultrasound-guided needle release of the A1 pulley alone.

**Methods:**

A total of 49 patients (55 fingers, thumb) with trigger fingers were included in this retrospective study. Twenty-seven fingers were treated with ultrasound-guided needle release of the A1 pulley alone (monotherapy group), and 28 fingers were treated with needle release of the A1 pulley combined with corticosteroid injection (combination group). Visual analog scale (VAS), Froimson scale, postoperative recurrence rate, and thickness of A1 pulley at baseline, Week-2, Week-12, and Month-6 were recorded.

**Results:**

Higher clinical cure rates were observed in the combination group at Week-2 after treatment among patients with the Froimson scale Grade III and IV (*p* < 0.05). Among Froimson scale Grade IV patients, the combination group had a significantly thinner thickness of A1 pulley and better articular pain relief at Week-2 (all *p* < 0.05). No significant differences were found in the clinical cure rate, the thickness of the A1 pulley, articular pain relief, and recurrence rate between the two groups at Week-12 and Month-6 (all *p* > 0.05).

**Conclusions:**

Ultrasonography-guided needle release of A1 pulley plus corticosteroid injection was superior to ultrasonography-guided A1 pulley needle release alone during early-stage treatment of severe patients with trigger fingers. Moreover, ultrasonography-guided A1 pulley needle release combined with corticosteroid injection narrows the thickness of the A1 pulley. It is necessary to carry out preoperative evaluation and individualized treatment for patients of various severities.

## Background

Trigger finger is a common clinical disorder that occurs pain, stiffness, and a sensation of locking or catching when the digit is compressed and extended [1]. Then bring about secondary inflammation and retinacular sheath hypertrophy. The condition has a lifetime risk of 2.6% in the general population and 10% in diabetics [2]. The cause of the trigger finger is the thickening of the A1 pulley due to repeating friction between the flexor tendon and the corresponding inner layer of the A1 pulley, or failure in prompt treatment of palm skin injury [3]. The normal A1 pulley has a mean thickness of 0.5 mm (range 0.4–0.6 mm), and the thickening of the A1 pulley for trigger fingers has a mean thickness of 1.8 mm (range 1.1–2.9 mm) [4]. The thickness of the A1 pulley and the flexor tendon is related to the severity of the trigger. When the A1 pulley becomes inflamed or thickened, the flexor tendon has a hard time gliding smoothly and allowing your finger to bend and flex [5]. The best treatment for the patient depends on the severity of the condition and whether patients have other contributing health factors. Generally, mild cases are first treated conservatively, with oral anti-inflammatory drugs, physical therapy, night splints, or corticosteroid injections; while severe cases are often treated with the percutaneous and open surgical release [6].

Modern ultrasonography-guided needle release of A1 pulley has been widely used for the treatment of trigger finger, normally the finger anatomic structures can be clearly gained and pulleys tendons can be fully analyzed [7]. Nikolaou et al. determine the efficacy of ultrasonography-guided A1 pulley release in their study, showing that using ultrasonography had better treatment than the open surgery technique [8]. The use of corticosteroid injection has proven to be an effective treatment since its introduction in 1953 [9], which is pursued first-line treatment for trigger finger due to it being less invasive and more cost-effective [10]. In addition, ultrasound-guided needle release of the A1 pulley with corticosteroid injection approaches had treatment benefits for the trigger finger. Our previous studies found that the excellent and success rate was 83.3% after treated ultrasound-guided needle release of the A1 pulley with corticosteroid injection [11]. However, side effects of steroid injection include pain, bleeding, steroid flare reaction, infection, and so on [12]. It is believed that due to corticosteroid injection cannot reverse the changes of chondroid metaplasia that take place at the A1 pulley, which is less effective with longstanding disease (> 6 months duration), diabetes mellitus, and multiple digit involvements [3]. It remains unclear whether ultrasound-guided needle release of A1 pulley alone obtains the same effectiveness as ultrasound-guided needle release of A1 pulley combined with corticosteroid injection. Therefore, a retrospective study was conducted to further evaluate the clinical effectiveness of ultrasonography-guided needle release of A1 pulley combined with corticosteroid injection by comparing it with ultrasound-guided needle release of the A1 pulley alone.

## Materials and methods

### Patients

From January 2018 to June 2019, 84 patients were diagnosed with trigger finger (thumb) in the Orthopedics Department of our hospital, of which 49 patients (55 fingers) were included in the retrospective study. Twenty-eight fingers underwent ultrasound-guided needle release of the A1 pulley combined with corticosteroid injection (combination group), and 27 fingers underwent single ultrasonography-guided needle release of the A1 pulley (monotherapy group). This study was approved by the ethical committee of the local hospital and informed consent was obtained from each patient.

The inclusion criteria were: (1) Patients aged between 18 and 80 years; (2) Patients who presented with typical symptoms of pain, tenderness or palpable nodules, and limited flexion and extension of the metacarpophalangeal joints of the fingers for more than 1 month, and had failed to previous conservative treatment such as splinting; (3) Ultrasonography presented with thickening of A1 pulley with or without flexor tendon swelling. The exclusion criteria were: (1) Patients with active rheumatoid arthritis, or other connective tissue diseases; (2) Patients who have previously undergone open surgery, percutaneous trigger thumb release or corticosteroid injection at the same site; (3) Patients with corticosteroid contraindication or intolerance to drug injection; (4) Patients with the poor general condition; (5) Pregnant patients; (6) Patients who lost regular follow-up; (7) Children. Considering the particularity of pediatric trigger finger, 60% of children could recover through splinting, surgery can be selected when deformity persists, and glucocorticoid injection is not recommended for children.

### Surgical procedures

**Fig. 1:**
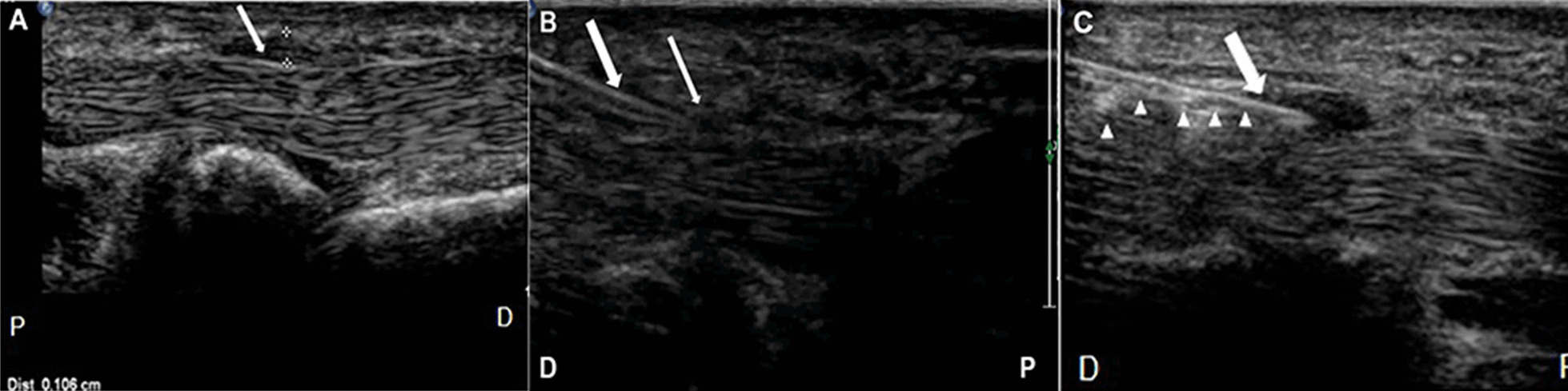
**A** A1 pulley thickening and the measurement method of thickness of A1 pulley (arrowhead). **B** Ultrasonography-guided needle release of A1 pulley. Thick arrow: needle head; Thin arrow: thickened A1 pulley. **C** Ultrasonography-guided corticosteroid injection in A1 pulley. Arrow: needle head; Triangle arrow: drug diffused in the tendon sheath. P: proximal end; D: distal end

Ultrasonic examinations were performed by using the Philips Elite scanner (Philips Healthcare Solutions, frequency 7–15 MHz) with the examination condition presetting to musculoskeletal mode. The patient was seated opposite the physician with the hand resting on a table, palm upwards, fingers pointing to the physician. After placing the probe at the metacarpophalangeal joint, the flexor tendon was cut longitudinally and the thickness of the A1 pulley was measured (Fig. 1A). Each measurement was repeated three times, and the mean value was quantified. The flexion and extension of the flexor tendon and whether it adhered to surrounding soft tissues were then observed dynamically.

The needle was inserted from the distal end to the proximal end, and the insertion point was selected at 1 cm from the distal end of the A1 pulley. Iodophor was applied to the sterile sleeve and skin to serve as a couplant. After routine skin disinfection with the needle entry point as the center, the sterile probe sleeve was covered on the probe coated with couplant. Local anesthesia was performed with 1 ml 2% lidocaine injection around the needle entry point by using a 1 ml syringe mounted with 21G needle. Then a new 1 ml syringe was loaded with 0.5 ml compound betamethasone injection. Inject the 21G needle quickly at a 90° angle. The 21G needle was inserted into the imaging plane by in-plane needle insertion. When the tip of the needle reached the A1 pulley, the A1 pulley was repeatedly punctured, and the body and tip of the needle were dynamically observed on the ultrasonic display screen (Fig. 1B), and then the drug was injected (Fig. 1C). During the trigger finger ultrasonography-guided release of the A1 pulley, patients were asked to perform active flexion and extension of the affected finger to test for any residual triggering. The complete release was confirmed by the disappearance of a grating sound and a full active range of motion after the end of the procedure. The needle was then withdrawn, the needle entry point was pressed for 3–5 min, and a small adhesive bandage was placed over the needle entry point. The wound had to be free from water contact for 48 h to prevent infection. Same procedures were performed in the monotherapy group until the finish of the ultrasonography-guided needle release of the A1 pulley, and the compound betamethasone injection was not injected. No additional medication was given in the two groups after the needle release of the A1 pulley. All the above operations were performed by a sonographer with 10 years’ experience in musculoskeletal ultrasound and interventional therapy. Whether to receive ultrasonography-guided needle release of A1 pulley combined with corticosteroid or ultrasound-guided needle release single was based on the clinician’s judgment at the time and the willingness of the patient.

### Clinical evaluation

The clinical symptoms were evaluated by using articular pain, the function of the joint, and the thickness of the A1 pulley. A ten-inch visual analog scale (VAS) ruler [13] was performed to evaluate the articular pain. The scale of VAS had marking every inch from 0 to 10 and the patients were told that an articular pain score of 0 represented no pain and 10 represented extreme pain. The function of the joint was assessed using the Froimson scale [14]: Froimson scale Grade I represented pain at the base of the digit, history of triggering, but not at presentation; Froimson scale Grade II represented triggering at presentation, unrestricted movement of the involved digit; Froimson scale Grade III represented continuous triggering, which is usually correctable by manipulation with the other hand; Froimson scale Grade IV represented total locking of the involved digit. After treatment, complete disappearance of clinical symptoms and Froimson scale Grade 1 was defined as a clinical cure. Patients with no pain, stiffness, and a sensation of locking or catching when the digit was compressed and extended, or mild pain with or without intermittent snapping and finger can be straightened with no flexion and lock state, reached a clinical cure. Recurrence within 6 months was defined as treatment failure. The thickness of the A1 pulley was measured by ultrasound. The normal A1 pulley had a mean thickness of 0.5 mm (range 0.4–0.6 mm), and the thickening of the A1 pulley for trigger fingers had a mean thickness of 1.8 mm (range 1.1–2.9 mm) [4]. Questionnaire collection and A1 pulley measurement were conducted by two sonographers with more than 5 years experience in musculoskeletal ultrasound at baseline, Week-2, Week-12, and Month-6.

### Statistical analysis

Data analyses were performed by using SPSS software, version 21.0 (SPSS Inc, Chicago, IL). Quantitative data (age, mean duration of disease, articular pain score, and the thickness of A1 pulley) were expressed as means ± standard deviations (SD) and were compared using Student’s t-test. Qualitative data (sex, lesion site, digit, grade of trigger finger, and clinical cure) were expressed as number and percentage and were compared using χ^2^ test. A *p* < 0.05 was considered statistically significant.

## Results

### Patients’ demographics

49 patients (55 fingers) were included in our study with a mean age of 55.13 ± 12.64 years (range 38–79 years). The mean duration of disease was 6.36 ± 2.72 months (range 2–12 months). Among these patients, 28 fingers underwent ultrasound-guided needle release of the A1 pulley combined with corticosteroid injection (combination group) and 27 fingers underwent single ultrasonography-guided needle release of A1 pulley (monotherapy group). The most frequent involved digit was thumb (71.43%) followed by index (14.29%), middle (10.71%) and ring fingers (3.57%). The baseline characteristics of two groups are shown in Table 1. The two groups were balanced in terms of age, sex, mean duration of disease, lesion site, digit involved and Grade of trigger finger (all *p* > 0.05).

**Table 1:** Baseline characteristics in two groups

	Combination group (n = 28)	Monotherapy group (n = 27)
Age (years, mean ± SD)	55.64 ± 13.47	56.44 ± 9.39
Sex (female/male, n/%)	19 (67.86)/9 (32.14)	19 (70.37)/8 (29.63)
Mean duration of disease (months, mean ± SD)	6.64 ± 2.90	5.78 ± 2.33
Lesion site (right/left hand, n/%)	20 (71.43)/8 (28.57)	16 (59.26)/11 (40.74)
Femural condylar	19	13
Femoral shaft	18	13
Digit involved (n, %)		
Thumb	20 (71.43)	20 (74.07)
Index	4 (14.29)	3 (11.11)
Middle	3 (10.71)	3 (11.11)
Ring	1 (3.57)	1 (3.71)
Small	0 (0.00)	0 (0.00)
Grade of trigger finger (n, %)		
Grade I	0 (0.00)	0 (0.00)
Grade II	7 (25.00)	6 (22.22)
Grade III	11 (39.29)	10 (37.04)
Grade IV	10 (35.71)	11 (40.74)

*SD* standard deviations

### Clinical evaluation outcomes

The improvements in clinical symptoms in Week-2, Week-12, and Month-6 are shown in Table 2. From baseline to Week-2, the clinical cure of patients with Froimson scale Grade III (32.14% vs. 3.70%, p < 0.05) and Grade IV (14.29% vs. 0.00%, p < 0.05) in the combination group were significantly higher than that in the monotherapy group. There was no significant difference between the two groups among patients with Froimson scale Grade II during the 6-month follow-up (all *p* > 0.05). In the 6-month follow-up, a total of 17 patients (62.96%) in the monotherapy group and 19 patients (67.86%) in the combination group reached clinical cure.

**Table 2:** The clinical symptoms improvement of the two groups at Week-2, Week-12, and Month-6

Grade	Improvement of symptoms	Combination group (n = 28)			Monotherapy group (n = 27)		
		Week-2	Week-12	Month-6	Week-2	Week-12	Month-6
Grade 2	Clinical cure (n, %)	6 (21.43)	6 (21.43)	6 (21.43)	4 (14.81)	5 (18.52)	5 (18.52)
Grade 3	Clinical cure (n, %)	9 (32.14)	9 (32.14)	9 (32.14)	1 (3.70)*	8 (29.63)	9 (33.33)
Grade 4	Clinical cure (n, %)	4 (14.29)	4 (14.29)	4 (14.29)	0 (0.00)*	3 (11.11)	3 (11.11)

^*^p < 0.05

There was no significant difference in VAS score between the two groups before treatment (*p* > 0.05). The combination group had a lower VAS score than the monotherapy group in Week-2 (2.00 ± 1.28 vs. 4.82 ± 1.64, *p* < 0.05). However, the VAS scores of the two groups were not statistically different at Week-12 and Month-6 after treatment (*p* > 0.05) (Table 3).

**Table 3:** Comparison of pain score before and after treatment between the two groups

VAS	Combination group (n = 28)	Monotherapy group (n = 27)
Baseline (mean ± SD)	5.89 ± 1.57	6.22 ± 1.25
Week-2 (mean ± SD)	2.00 ± 1.28	4.82 ± 1.64^*^
Week-12 post-treatment (mean ± SD)	1.39 ± 1.57	2.19 ± 1.57
Month-6 post-treatment (mean ± SD)	1.25 ± 1.46	0.96 ± 1.53

*VAS* visual analogue scale, *SD* standard deviations

^*^p < 0.05

The ultrasound results showed that the thickness of the A1 pulley at Week-2 in the combination group was thinner than that in the monotherapy group (0.77 ± 0.30 vs. 1.20 ± 0.53 mm, *p* < 0.05). However, the thickness of the A1 pulley was not statistically different between the two groups at Week-12 and Month-6 (all *p* > 0.05) (Table 4).

**Table 4:** Comparison of thickness of A1 pulley (mm) before and after treatment between the two groups

	Combination group (n = 28)	Monotherapy group (n = 27)
Pre-treatment	1.51 ± 0.50	1.69 ± 0.56
Week-2 post-treatment	0.77 ± 0.30	1.20 ± 0.53*
Week-12 post-treatment	0.51 ± 0.24	0.75 ± 0.43
Month-6 post- treatment	0.39 ± 0.22	0.48 ± 0.35

^*^p < 0.05

### Recurrence rate and complication

In this study, 7 cases (25.92%) in monotherapy group and 6 cases (21.43%) in combination group relapsed within 6 months. There was no significant difference in the treatment failure rate between the two groups (*p* > 0.05). No serious complications occurred in both groups.

## Discussion

In this study, we compared the therapeutic effect of ultrasonography-guided needle release of the A1 pulley alone and ultrasonography-guided needle release of the A1 pulley combined with corticosteroid injection for the trigger finger. Our results found that the clinical cure of patients with Froimson scale Grade III (32.14% vs. 3.70%, p < 0.05) and Grade IV (14.29% vs. 0.00%, p < 0.05) in the combination group were significantly higher than that in the monotherapy group at Week-2, and combination group had lower VAS score than monotherapy group (2.00 ± 1.28 vs. 4.82 ± 1.64, p < 0.05). There were no significant differences in clinical efficacy between the two groups at Week-12 and Month-6 and patients with mild disease. It showed that the combination therapy may be more effective than ultrasonography-guided needle release of the A1 pulley alone during early-stage treatment of severe patients with trigger fingers, and the combination therapy is superior to the single needle release in early-stage articular pain relief.

The outcomes reported in this study are relatively fair (62.9–67.8% clinical cure rate) compared with either report that used open and percutaneous release. Previous studies have shown that the cure rate after open surgical release of the A1 pulley varies from 60 to 97% [2]. Lapègue et al. proved that ultrasonography-guided percutaneous treatment of the trigger finger by releasing the A1 pulley with a cure rate of 81.7% [7]. However, our clinical cure rate in this study is lower than the previous report using ultrasound-guided A1 pulley release. Bodor et al. found ultrasound-guided A1 pulley combined with triamcinolone have an effect with a 94% cure rate at 6 months for trigger finger [15]. The possible reason was that we used different corticosteroids (betamethasone vs. triamcinolone [15]) and included more thumbs (71.43% vs. 48% [15]) in the sample. The pathophysiology of trigger finger is that the system undergoes inflammatory and hypercellular changes to affect the normal motion [16]. Triamcinolone is a potent anti-inflammatory corticosteroid which beneficial for reducing cellular accumulation at inflammatory sites [17]. Ketchum et al. found that triamcinolone was more effective than betamethasone in treating chronic inflammation, such as trigger fingers [18]. Due to the vast differences in the number of thumbs and fingers studied in each report may potentially, the thumb and the finger had different anatomic properties and the thumb was more difficult to treat, which contributed to exclusion bias and may affect the results [19]. Another reason was that we included more severe patients (78% vs. 10%), causing clinical treatment more complicated and difficult.

There are some striking findings of the present study when we compared ultrasonography-guided A1 pulley needle release plus corticosteroid injection with ultrasonography-guided needle release alone for the trigger finger. The results showed that patients with Froimson scale Grade III and IV had a higher clinical cure rate in the combination group at 2 weeks after treatment, suggesting the early combined treatment had better efficacy than simple needle therapy only in patients with severe symptoms (Grade III and IV). Early combined treatment has not shown an obvious advantage in patients with mild symptoms (Grade II) and the medium and long-term efficacy. The possible reason is that steroid injection increases the patients’ possible untoward effects such as pain, bleeding, steroid flare reaction and infection, which is more obvious in mild symptoms degree [20]. Therefore, it is necessary to carry out preoperative evaluation and individualized treatment for patients of various severities. Moreover, we found combination group had better articular pain relief than the monotherapy group at 2 weeks postoperatively, and no significant differences in 12 weeks and 6 months. Showed the superiority of combined treatment over the single needle release in early-stage articular pain relief. It may be related to steroid injection is not superior to ultrasonography-guided needle release in articular pain relief clinical efficacy, especially in the long term. Anderson et al. showed that pain recurrent episodes occurred in 27% after corticosteroids injection after a mean symptom-free interval of 11.3 months [21]. In addition, the ultrasound results showed that the thickness of the combination group 2 weeks postoperatively was thinner than the monotherapy group (p < 0.05), suggesting the effectiveness of these combination group treatment methods. It may be related to the corticosteroids decreasing inflammation in the treatment of trigger finger [22].

Attention should be paid to the following points during our procedure: (1) Aseptic surgical operation is important and necessary. (2) The surgeon should have adequate training and expertise in the anatomical structure of the finger, control the depth and stability of the hypodermic syringes, so that the depth of needle tip reach between tendon and A1 pulley. (3) The ulnar artery, ulnar nerve, radial artery and radial nerve should be verified before the needle puncture. Attention should be paid to avoid damaging the median nerve, radial and ulnar sides of nerve and blood vessels during the process of needle puncture. (4) When a sense of falling out occurred during acupuncture, the pulley may have loosened. Patient may be asked to perform finger flexion and extension activities to understand the degree of release. If the movement was normal and the sound disappeared, the A1 pulley has been completely loosened. Then inject the drug.

There were several limitations. Firstly, this study was a retrospective study conducted at a single institute which had certain limitations in clinical application. Secondly, our study was limited in the number of patients; a larger group of patients would probably have strengthened the results. Additionally, further studies with large samples are needed to elucidate the long-term clinical outcomes.

## Conclusions

This technique of ultrasound-guided needle release of A1 pulley plus corticosteroid injection is an effective procedure in the treatment of trigger fingers. In the early-stage treatment of severe patients with trigger fingers, ultrasonography-guided needle release of A1 pulley plus corticosteroid injection was superior to ultrasonography-guided A1 pulley needle release alone. There were no significant differences in clinical efficacy between patients with mild disease in long-term treatment. Therefore, it is necessary to carry out preoperative evaluation and individualized treatment for patients of various severities.

## Data Availability

All data generated or analysed during this study are included in this published article.
